# Altered brain arginine metabolism in schizophrenia

**DOI:** 10.1038/tp.2016.144

**Published:** 2016-08-16

**Authors:** P Liu, Y Jing, N D Collie, B Dean, D K Bilkey, H Zhang

**Affiliations:** 1 Department of Anatomy, University of Otago, Dunedin, New Zealand; 2 Brain Health Research Centre, University of Otago, Dunedin, New Zealand; 3 The Molecular Psychiatry Laboratory, The Florey Institute for Neuroscience and Mental Health, Parkville, VIC, Australia; 4 Department of Psychology, University of Otago, Dunedin, New Zealand; 5 School of Pharmacy, University of Otago, Dunedin, New Zealand

## Abstract

Previous research implicates altered metabolism of l-arginine, a versatile amino acid with a number of bioactive metabolites, in the pathogenesis of schizophrenia. The present study, for we believe the first time, systematically compared the metabolic profile of l-arginine in the frontal cortex (Brodmann's area 8) obtained post-mortem from schizophrenic individuals and age- and gender-matched non-psychiatric controls (*n*=20 per group). The enzyme assays revealed no change in total nitric oxide synthase (NOS) activity, but significantly increased arginase activity in the schizophrenia group. Western blot showed reduced endothelial NOS protein expression and increased arginase II protein level in the disease group. High-performance liquid chromatography and liquid chromatography/mass spectrometric assays confirmed significantly reduced levels of γ-aminobutyric acid (GABA), but increased agmatine concentration and glutamate/GABA ratio in the schizophrenia cases. Regression analysis indicated positive correlations between arginase activity and the age of disease onset and between l-ornithine level and the duration of illness. Moreover, cluster analyses revealed that l-arginine and its main metabolites l-citrulline, l-ornithine and agmatine formed distinct groups, which were altered in the schizophrenia group. The present study provides further evidence of altered brain arginine metabolism in schizophrenia, which enhances our understanding of the pathogenesis of schizophrenia and may lead to the future development of novel preventions and/or therapeutics for the disease.

## Introduction

Schizophrenia is a chronic mental disorder characterised by positive symptoms (for example, hallucinations, delusions and thought disorder), negative symptoms (for example, deficits in social interaction, emotion and motivation) and cognitive dysfunction (for example, impairments of attention and working memory). Although the biological basis of schizophrenia is poorly understood, a number of factors, such as dopamine hyperfunction, glutamatergic hypofunction, GABAergic deficits, cholinergic system dysfunction, stress vulnerability and neurodevelopmental disruption, have been linked to the aetiology and/or pathophysiology of the disease.^1, 2, 3, 4, 5^ Recently, altered arginine metabolism has also been implicated in the pathogenesis of schizophrenia.^6, 7, 8, 9^

l-arginine, a versatile semi-essential amino acid, is widely distributed in the brain. It can be metabolised to produce a number of bioactive molecules, for example, nitric oxide (NO) and l-citrulline by nitric oxide synthase (NOS), l-ornithine and urea by arginase, and agmatine by arginine decarboxylase.^10^ The gaseous signalling molecule NO, which can be derived from constitutive neuronal and endothelial NOS (nNOS and eNOS, respectively), regulates synaptic plasticity, neurotransmitter release, neurodevelopment and cerebral blood flow.^11, 12, 13, 14^ In contrast, excessive amounts of the free radical NO, derived from inducible NOS (iNOS), lead to neurotoxicity and neurodegeneration.^15^ l-ornithine, another l-arginine metabolite, is the main precursor of polyamines putrescine (through ornithine decarboxylase), spermidine and spermine, which are essential for maintaining normal cellular function.^11, 12, 13, 14^ l-orthinine can also be channelled to produce glutamate, glutamine and γ-aminobutyric acid (GABA).^10^ In addition, the l-arginine metabolite agmatine is a novel putative neurotransmitter and interacts with a number of receptor subtypes, including n-methyl-d-aspartate receptors.^16, 17, 18^ It inhibits nNOS and iNOS, but stimulates eNOS, regulates ornithine decarboxylase and can itself be converted into putrescine by agmatinase.^16^ Hence, agmatine has an important role in regulating NO and polyamine production.

In the last decade, several studies have reported alterations of arginine metabolism in schizophrenia, and evidence accrued implicates polyamines in the aetiology and pathology of several mental disorders, including schizophrenia.^6^ For example, research has shown elevated NO and NOS expression levels in the brain and plasma samples of schizophrenic individuals.^19, 20, 21^ Human genetic and metabolomics studies have identified schizophrenia risk genes encoding nNOS (NOS1) and several downstream effectors of nNOS,^7, 22, 23, 24^ and reported disturbed biosynthetic and signalling pathways linked to arginine metabolism^25, 26^ and reduced expression in genes involved in the regulation of l-ornithine and polyamine metabolism.^26^ Other studies have demonstrated decreased plasma arginase activity in schizophrenic patients,^9^ and a positive correlation between the serum levels of l-ornithine and the duration of illness.^27^ More recently, a significant increase in agmatine in the plasma was found in schizophrenic patients,^8^ suggesting blood agmatine as a potential biomarker of the disease.

Whereas the findings presented above suggest that altered arginine metabolism may have a critical role in the pathogenesis of schizophrenia, this hypothesis is based on the information obtained from studies of a single metabolic pathway. Given the parallel pathways involved in arginine metabolism, it is essential that we understand how the brain arginine metabolic profile changes in schizophrenic individuals. Although increased blood agmatine level has been found in patients with schizophrenia and can potentially be a biomarker of the disease,^8^ it is currently unknown how the agmatine level changes in the diseased brains. The present study was, therefore, designed to systematically compare the activity and protein expression of arginine metabolic enzymes, as well as the tissue concentrations of l-arginine and its nine downstream metabolites in the post-mortem frontal cortex (Brodmann's area 8, BA8) of patients with schizophrenia and non-psychiatric controls. BA8 (the frontal eye field) is known to be responsible for eye tracking dysfunction, which is the most widely replicated behavioural deficit in schizophrenia (for review see Levy *et al.*^28^). Moreover, BA8 displays grey matter abnormalities and is also involved in prospective memory deficits in schizophrenia.^29, 30, 31^

## Materials and methods

### Human tissue samples

Brain tissue samples were obtained from the Victorian Brain Bank Network at the Florey Institute for Neuroscience and Mental Health. The collection and supply of tissue were approved by the Ethics Committee of the Victorian Institute of Forensic Medicine and the Tissue Access Committee of the Victorian Brain Bank Network, respectively. In the present study, unfixed frozen small blocks of grey matter of the left frontal cortex (BA8) from patients with schizophrenia (*n*=20) and controls with non-psychiatric conditions (*n*=20) were used (Table 1).

The brain tissue collection and storage were detailed previously.^32^ All cadavers were stored at 4 ^o^C within 5 h of death. During case history review, age at death, gender, post-mortem interval (PMI) and brain pH (a measure of the quality of tissue preservation) were determined.^33^ With regards to PMI, where death was witnessed, PMI was from the time of death to autopsy. If death had not been witnessed, PMI was taken as the mid point between the time the person was last observed alive and the time found dead (maximum of 5 h). For all of the cases of schizophrenia, the diagnosis was performed according to the Diagnostic and Statistical Manual of Mental Disorders 4th edition criteria following a review of clinical records using the Diagnostic Instrument for Brain Studies (a structured instrument allowing a consensus psychiatric diagnosis to be made after death), and was agreed by two senior psychiatrists and the psychologist completing the case history review.^34, 35^ The age of disease onset was noted, the duration of illness (DI) was calculated and the final recorded antipsychotic drug doses (FRADD; presented as chlorpromazine equivalents) and lifetime exposure to antipsychotic drugs (LEAP) were recorded. For all non-psychiatric cases, an extensive review of case histories, along with questioning of families and treating clinicians, was undertaken to exclude any history of psychiatric illness. The demographic characteristics of this study are shown in Table 1 and Supplementary Table 1.

### Tissue preparation

The frozen brain tissue (in small blocks) from each case was wrapped in tinfoil and broken mechanically. The tissue was then mixed and divided into two parts. Protease-inhibitory buffer containing 50 mM Tris-HCl (pH 7.4), 10 μM phenylmethylsulfonyl fluoride, 15 μM pepstatin A and 2 μM leupeptin (1:10 w/v) was added to the first part of tissue on ice, and the samples were then homogenised using ultrasonification (Branson Sonifier 150D, Branson Ultrasonics, Danbury CT, USA) and centrifuged at 12 000 *g* for 10 min at 4 ^o^C. Protein concentrations in the supernatant were measured based on the Bradford method^36^ using a Bio-Rad protein assay dye reagent concentrate and Bio-Rad Benchmark Plus microplate spectrophotometer (Bio-Rad Laboratories, Hercules, CA, USA).^37, 38, 39^ Each supernatant was then aliquoted and used for the enzyme assays and western blot analysis. The second part of tissue from each case was weighed, homogenised in ice-cold 10% perchloric acid (~50 mg wet weight per millilitre) and centrifuged at 10 000 r.p.m. for 10 min to precipitate protein. The supernatants (the perchloric acid extracts) were frozen immediately and stored at −80 ^o^C for later high-performance liquid chromatography and liquid chromatography/mass spectrometric assays. The experimenters were blind to the grouping information.

### NOS and arginase assays

A radioenzymatic assay technique^40^ was employed to determine total NOS activity by measuring the ability of tissue homogenates to convert [^3^H] l-arginine to [^3^H] l-citrulline in the presence of cofactors, and the contribution of iNOS (calcium-independent) to total NOS activity was assessed in the absence of calcium as described previously.^39, 41^ Regarding arginase activity, a spectrophotometric assay method was used by measuring the amount of newly formed urea from l-arginine, as detailed in our previous publications.^39, 41^ All assays were performed in triplicate. The samples from both groups were processed at the same time and the order was counterbalanced. NOS and arginase activities were expressed as pmol [^3^H] l-citrulline per 30 min per mg protein and μg urea per mg protein, respectively.^39, 41^

### Western blot analysis

The protein expression of NOS (nNOS and eNOS), arginase (arginase I and arginase II) and β-actin in each sample was determined using the western blot method as described previously.^39^ Briefly, the protein concentrations in all of the brain samples were equalized to 2 mg ml^−1^. The supernatants were mixed with gel loading buffer in a ratio of 1:1 and then boiled for 5 min. Ten microlitre of each sample was loaded in each well on a precast 4–12% Bis-Tris Criterion gel (Bio-Rad), and a pre-stained protein marker (41.5–203 kDa; Bio-Rad) was run on the same gel. The proteins were transferred overnight to polyvinylidene-difluoride membranes using a transblotting apparatus (Bio-Rad). Nonspecific IgG binding was blocked, and the membranes were then incubated with an affinity-purified monoclonal mouse antibody raised against nNOS (Santa Cruz Biotechnology, Dallas, TX, USA; sc-5302) or arginase I (sc-166920), or polyclonal rabbit antibody raised against eNOS (sc-653) or arginase II (sc-20151) overnight at 4 ^o^C. The secondary antibody was an anti-mouse (sc-2005) or an anti-rabbit (sc-2004) IgG linked to horseradish peroxidase. In order to ensure that the same amount of protein was loaded in each lane, an IgG monoclonal antibody against β-actin (sc-47778) was used as a loading control. Immunodetection was performed using the enhanced chemiluminescence system (Amersham, GE Healthcare Life Sciences, Little Chalfont, Buckinghamshire, UK). Hyperfilms (Amersham) were analysed using densitometry to determine the quantity of protein expressed in each group using the Bio-Rad Quantity One software. Results were expressed as volume of the band (optical density × area of the band), and normalised by the corresponding β-actin loading controls.

### Amino acid and polyamine analyses

Determination of amino acids (l-arginine, l-citrulline, l-ornithine, glutamate, glutamine and GABA) and polyamines spermidine and spermine were carried out using high-performance liquid chromatography, and agmatine and putrescine levels were measured by a highly sensitive liquid chromatography/mass spectrometric method, as we have previously described.^37, 38, 39, 41^ High-purity external and internal standards were used (Sigma, Sydney, NSW, Australia). All other chemicals were of analytical grade.

For agmatine and putrescine, the tissue concentrations were measured with the API 3200 liquid chromatography/mass spectrometric system (a fully integrated triple quadrupole mass spectrometer).^37, 38, 39, 41^ Briefly, after adding the internal standard (1,7-diaminoheptane) to 20 μl of the perchloric acid extracts, the samples were alkalized with saturated sodium carbonate and derivatized with dansyl chloride. Agmatine, putrescine and internal standard were extracted with toluene. The toluene phase was evaporated to dryness, reconstituted and injected on the liquid chromatography/mass spectrometric system. The samples were analysed using a reversed-phase C_18_ column (150 × 2.0 mm, 5 mm, Phenomenex, Lane Cove, NSW, Australia) with 80% acetonitrile: 20% water containing 0.1% formic acid as mobile phase at a flow rate of 0.2 ml min^−1^. The retention times of agmatine, putrescine and the internal standard were 1.7, 4.0 and 4.8 min, respectively. The total run time was 15 min. Detection with MS/MS used an electrospray interface in the positive ion mode. The standard curves for agmatine and putrescine were linear up to 1000 ng ml^−1^ (*R*^2^>0.99). The intra- and inter-day coefficients of variation were <15%.

The samples from both groups were assayed at the same time in a counterbalanced manner, and the assays were performed in triplicate. The concentrations of l-arginine and its nine downstream metabolites in tissue were calculated with reference to the peak area of external standards, and values were expressed as μg per g wet tissue.^37, 38, 39, 41^

### Statistical analysis

The difference between the control and schizophrenia groups for each neurochemical variable was analysed using an unpaired *t*-test or Mann–Whitney *U-*test, and significance was set at *P*<0.05 for all comparisons. Linear regression analysis was performed to determine how neurochemical levels in control or schizophrenia cases relate to age, PMI and brain pH (for both groups), as well as the age of disease onset, the duration of illness, FRADD and LEAP (for the schizophrenia group only). Owing to multiple comparisons, the level of significance was set at *P*⩽0.01 (equivalent to a Geisser–Greenhouse correction for potential violation of the assumption of sphericity^42^). All calculations were performed with the Prism statistics programme, and only the significant statistics are reported.

As we had measures of the tissue concentrations of l-arginine and its three main metabolites (l-citrulline, l-ornithine and agmatine), cluster analysis (an exploratory data analysis to sort different variables into groups) was performed using Minitab 16 for the control or schizophrenia group to determine which neurochemical variables co-varied.^37, 38^ Before the analysis, the data were standardized to obtain *z-*values. Agglomerative methods were then used on the correlation coefficient distance, which started with each observation as a cluster and then with each step observations were combined to form clusters until there was only one large cluster. Comparisons between different cluster analysis algorithms indicated that Complete linkage, McQuitty linkage, Average linkage and Ward linkage produced similar results (data not shown). Therefore, Ward linkage (based on the sum of squares between the two clusters, summed over all variables) was used for all of the analyses. Dendrograms were generated using Minitab 16 to display the groups formed by clustering of variables and their similarity levels (defined as percentage of the minimum distance at each step relative to the maximum intervariable distance).^37, 38^

## Results

### NOS and arginase activity and protein expression

**Figure 1:**
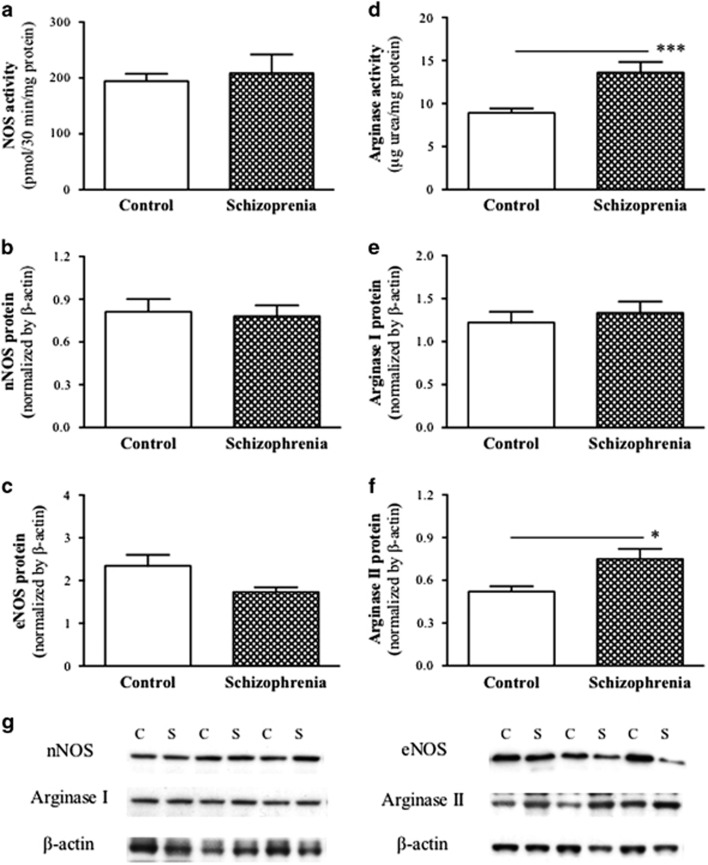
Mean (±s.e.m.) total NOS (**a**) and arginase (**d**) activity, and protein levels of nNOS (**b**), eNOS (**c**), arginase I (**e**) and arginase II (**f**) in the frontal cortex (BA8) from non-psychiatric control and schizophrenia cases (*n*=20 per group). (**g**) Example of western blots showing nNOS, eNOS, arginase I and arginase II protein expression between the control (**c**) and schizophrenia (s) groups with their corresponding β-actin loading controls. Asterisks indicate significant differences between groups at *P<0.05 or ***P<0.001. BA8, Brodmann's area 8; eNOS, endothelial NOS; NOS, nitric oxide synthase; nNOS, neuronal NOS.

The radioenzymatic assay revealed no significant difference in total NOS activity between the control and schizophrenia groups (*P*=0.86; Figure 1a). Western blot analysis indicated a reduced eNOS protein level (~25%) in the schizophrenia cases relative to the controls (*P*=0.045; Figures 1c and g), with no group difference in nNOS (*P*=0.86; Figures 1b and g) or β-actin (*P*=0.55, data not shown) protein expression. iNOS protein expression was not determined because of its undetectable activity in the absence of calcium and tissue availability.

The spectrophotometric assay revealed a significant increase in arginase activity (~52%) in the schizophrenia group when compared with the control group (*P*=0.0029; Figure 1d). Western blot analysis showed a 45% increase in arginase II protein in the schizophrenia group (*P*=0.02; Figures 1f and g), and there was no significant difference between groups in arginase I protein expression (*P*=0.58; Figures 1e and g).

### Tissue concentrations of l-arginine and its downstream metabolites

**Figure 2:**
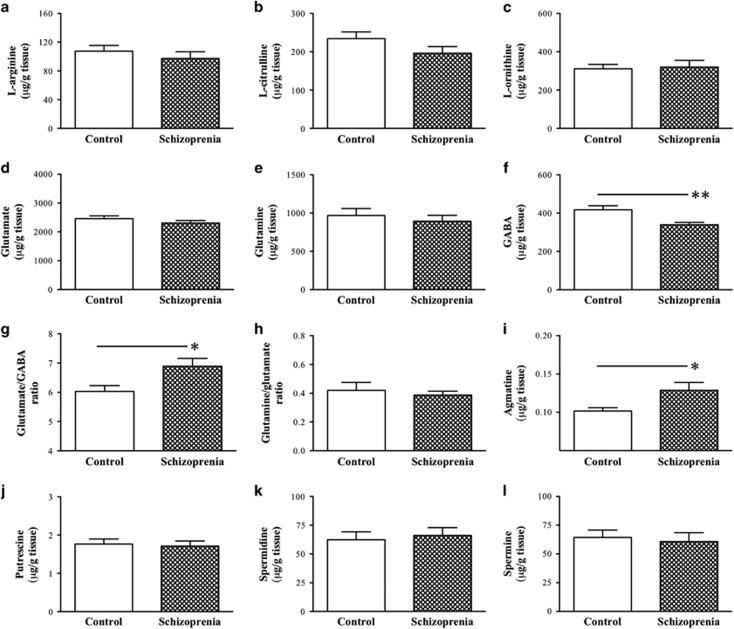
Mean (±s.e.m.) levels of l-arginine (**a**), l-citrulline (**b**), l-ornithine (**c**), glutamate (**d**), glutamine (**e**), GABA (**f**), glutamate/GABA ratio (**g**), glutamine/glutamate ratio (**h**), agmatine (**i**), putrescine (**j**), spermidine (**k**) and spermine (**l**) in the frontal cortex (BA8) from non-psychiatric control and schizophrenia cases (*n*=20 per group). Asterisks indicate significant differences between groups at *P<0.05 or **P<0.01. BA8, Brodmann's area 8; GABA, γ-aminobutyric acid.

High-performance liquid chromatography and liquid chromatography/mass spectrometric assays were performed to determine the concentrations of l-arginine and its nine downstream metabolites in the frontal cortex (BA8) from the schizophrenia and control cases. We found no significant difference between groups in l-arginine (Figure 2a), l-citrulline (Figure 2b), l-ornithine (Figure 2c), glutamate (Figure 2d), glutamine (Figure 2e), glutamine/glutamate ratio (Figure 2h), putrescine (Figure 2j), spermidine (Figure 2k) or spermine (Figure 2l). However, the GABA level was 20% lower (*P*=0.009; Figure 2f), the glutamate/GABA ratio was significantly higher (*P*=0.03, ~14% Figure 2g) and agmatine levels were higher (*P*=0.03, ~26% Figure 2i) in schizophrenia cases when compared with controls.

### Correlations with disease characteristics

**Figure 3:**
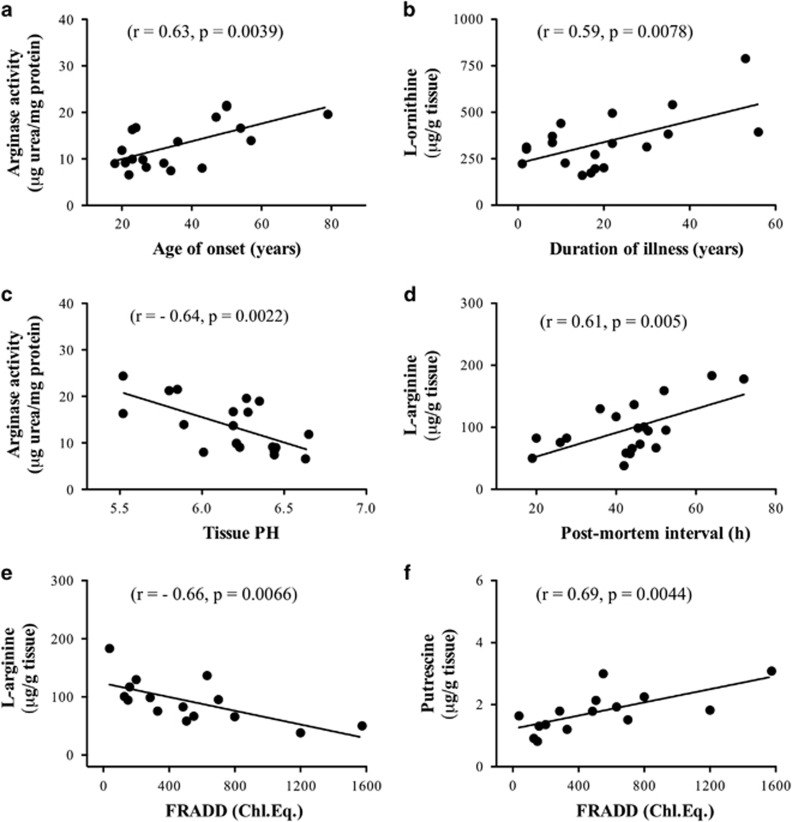
Scattergrams showing the significant correlations between arginase activity and the age of disease onset (**a**), l-ornithine and the duration of illness (**b**), arginase activity and brain tissue pH (**c**), l-arginine and post-mortem interval (**d**), l-arginine and FRADD (**e**), and putrescine and FRADD (**f**) across 15–20 schizophrenic cases. The dimensions of neurochemical levels were μg urea per mg protein for arginase activity and μg per g wet tissue for l-arginine, l-ornithine and putrescine. FRADD, final recorded antipsychotic drug.

Linear regression analysis revealed significant positive correlations between arginase activity and the age of onset (*r*=0.63, *P*=0.0039; Figure 3a), and between l-ornithine and the duration of illness (*r*=0.59, *P*=0.0078; Figure 3b) in the schizophrenia group. Because there were no significant correlations between age and arginine activity (*r*=0.35, *P*=0.13; data not shown) and l-ornithine (*r*=0.06, *P*=0.81; data not shown), the above-mentioned positive correlations are not simply the effects of age. We also found significant negative correlations between brain tissue pH and arginase activity (*r*=−0.64, *P*=0.0022; Figure 3c) and between FRADD and l-arginine (*r*=−0.66, *P*=0.0066; Figure 3e), and significant positive correlations between PMI and l-arginine (*r*=0.61, *P*=0.005; Figure 3d) and between FRADD and putrescine (*r*=0.69, *P*=0.0044; Figure 3f) in the schizophrenia group.

### Cluster analyses

**Figure 4:**
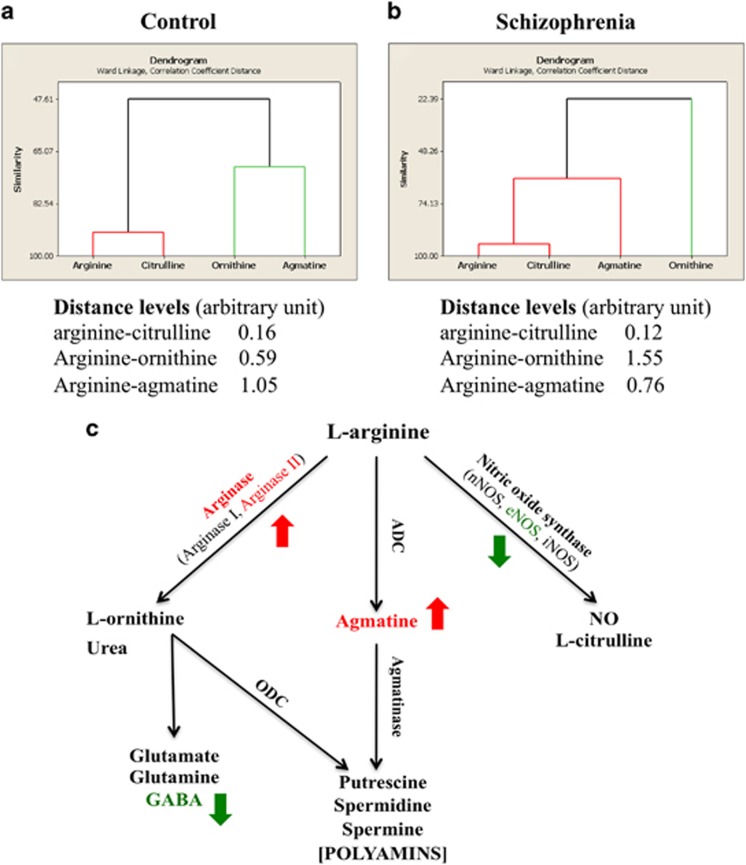
Dendrograms showing the similarities in the degree of expression of l-arginine (arginine) and its three main metabolites l-citrulline (citrulline), l-ornithine (ornithine) and agmatine in the frontal cortex (BA8) from non-psychiatric control (**a**) and schizophrenia (**b**) cases (*n*=20 per group). The numbers indicate the distance levels (arbitrary unit) between arginine and each metabolite. (**c**) Arginine metabolic pathways. l-arginine can be metabolised by NOS, arginase and ADC to form a number of bioactive molecules (see the Introduction for detailed description). We found increased levels of arginase activity, arginase II protein expression and agmatine tissue concentration (indicated by the red letters and arrows), and reduced eNOS protein expression and GABA level (indicated by the green letters and arrows) in the schizophrenia cases. ADC, arginine decarboxylase; BA8, Brodmann's area 8; eNOS, endothelial NOS; GABA, γ-aminobutyric acid; iNOS, inducible NOS; NO, nitric oxide; NOS, nitric oxide synthase; nNOS, neuronal NOS.

We further analysed how l-arginine formed clusters with its three main metabolites l-citrulline, l-ornithine and agmatine in each group. In the control group, l-arginine and l-citrulline formed one cluster, whereas l-ornithine and agmatine formed another (Figure 4a). In the schizophrenia group, however, l-arginine, l-citrulline and agmatine together formed one cluster with l-ornithine alone as another (Figure 4b). When the cluster distance (arbitrary unit) between l-arginine and its three main metabolites in each group were compared, in the control group l-arginine had the shortest distance to l-citrulline (0.16), followed by l-ornithine (0.59) and agmatine (1.05; Figure 4a). In the schizophrenia group, however, although l-arginine still had the shortest distance to l-citrulline (0.12), it was far away from l-ornithine (1.55) but became closer to agmatine (0.76; Figure 4b). These findings demonstrate that the relationship between l-arginine and its three main metabolites is altered in region BA8 of patients with schizophrenia.

## Discussion

To the best of our knowledge, the present study provides the first description of the metabolic profile of l-arginine in the frontal cortex (BA8), taking into account its parallel NOS, arginase and arginine decarboxylase pathways in patients with schizophrenia compared with their age- and gender-matched non-psychiatric control cases. Because tissue samples were well-matched, there being no significant differences between groups in the PMI and brain tissue pH, altered arginine metabolism in BA8 of patients with schizophrenia observed should be attributed to the disease, rather than the quality of tissue.

Earlier research has identified schizophrenia risk genes that encode nNOS and several downstream effectors of nNOS.^7, 22, 23, 24^ However, we found no changes in total NOS activity (with undetectable iNOS activity) and nNOS protein expression, but reduced eNOS protein levels, in BA8 of patients with schizophrenia. As eNOS has an important role in maintaining the cerebral blood flow,^6^ the decreased eNOS protein level may, to some extent, contribute to the reported cerebral blood flow reduction in the frontal cortex in schizophrenia.^43, 44, 45^ An earlier study reported decreased plasma arginase activity in patients with schizophrenia.^9^ The present study, interestingly, found an over 50% increase in arginase activity in BA8 accompanied by a significant upregulation of arginase II in the schizophrenia group. It is currently unclear how arginase changes in blood correlate with those in the brain tissue.

The present study further determined how the tissue concentrations of l-arginine and its nine downstream metabolites in BA8 changed in schizophrenia. We found no changes in l-arginine, l-citrulline and l-ornithine; however, there were reduced GABA levels and increased glutamate/GABA ratios in the schizophrenia group, which appear to be consistent with GABAergic deficits seen in schizophrenia.^1, 46, 47^ Although earlier work showed increased^46^ or decreased^48^ glutamate levels in the frontal cortex of schizophrenia patients, we found no significant change in glutamate between the control and schizophrenia groups (see also Korpi *et al.*^49^). Thus, the increased glutamate/GABA ratio in the schizophrenia group is largely due to the reduction of GABA, supporting the prominent role of GABAergic deficits in the aetiology and/or pathophysiology of schizophrenia.

Polyamines and agmatine have also been implicated in psychiatric disorders.^6^ Middleton *et al.*^26^ reported reduced expression in genes involved in the regulation of l-ornithine and polyamine metabolism in the dorsal prefrontal cortex of patients with schizophrenia. However, the present study did not detect differences in putrescine, spermidine or spermine in BA8 between the control and schizophrenia groups, which is consistent with an earlier study that failed to detect changes in polyamines in the hippocampus and frontal cortex in schizophrenia.^50^ It is of interest to note that we found significantly increased agmatine level in BA8 in schizophrenia patients. Agmatine is considered to be a novel putative neurotransmitter,^16, 17^ and directly participates in learning and memory processing.^51, 52, 53, 54, 55^ At synapses, it is colocalised with glutamate,^56, 57, 58^ and evokes a non-competitive voltage- and concentration-dependent block of the n-methyl-d-aspartate receptors.^17, 59^ Hence, agmatine may have a critical role in controlling glutamate-mediated excitatory moiety. To this end, increased agmatine level may potentially lead to glutamatergic hypofunction,^60^ although the question of how synaptic agmatine changes in schizophrenia is currently unclear. Interestingly, high dose of agmatine has been shown to disrupt prepulse inhibition of acoustic startle reflex, an operational measure of sensorimotor gating (a benchmark test for schizophrenia^61^), in rats.^62^ In the present study, we were unable to investigate how arginine decarboxylase (the biosynthesis enzyme of agmatine) changed in schizophrenia, as fresh tissue is required for measuring its activity.^63^ Bernstein *et al.*^64^ reported strongly upregulated agmatinase, which converts agmatine to putrescine, in the hippocampus of patients with mood disorders (unipolar and bipolar depression). It is therefore necessary to investigate how agmatine is affected in schizophrenia at the subcellular levels, and how its biosynthesis (arginine decarboxylase) and degradation (agmatinase) enzymes change in the brains of schizophrenic individuals in the future.

It is of interest to note that the plasma agmatine level was increased over threefold in schizophrenic patients relative to healthy controls, and a receiver operating characteristic curve analysis revealed the predictive value of plasma agmatine in differentiating patients with schizophrenia from healthy controls.^8^ The findings of this study suggest that the blood agmatine level may be a potential biomarker of schizophrenia. Since the present study and the study of Uzbay *et al.*^8^ have both shown increased agmatine levels in schizophrenic patients, the findings merit future investigations into whether excess agmatine contributes to the development of the disease. On the other hand, however, there is also a need to determine whether this change is a compensatory process to normalise the alterations in the NOS and arginase–polyamine pathways, given the role of agmatine in regulating the NO and polyamine production.

The present study measured the levels of l-arginine and its three main metabolites l-citrulline, l-ornithine and agmatine in BA8 in both the control and schizophrenia groups, which allowed us to perform cluster analysis to determine whether the four inter-related neurochemical variables clustered into distinct groups and whether the pattern changed as a function of the disease. Under physiological condition, NOS is the predominant pathway, as it has an ~1000-fold greater affinity for l-arginine than arginase,^10^ and endogenous agmatine levels in the mammalian brain are low.^63^ We found that l-arginine was close to l-citrulline in the control group; however, it became closer to agmatine in the schizophrenia group, indicating an altered relationship between l-arginine and its three main metabolites in schizophrenic brains. We also observed significant positive correlations between arginase activity and the age of disease onset and between l-ornithine and the duration of schizophrenia, suggesting that these neurochemical changes may have aetiological and/or functional significance.

There are some limitations in the present study. First, our control and schizophrenia cohorts contained a highly heterogeneous population, with large variations in drug histories in schizophrenia cases (see Supplementary Table 1). Regression analysis revealed that the FRADDs correlated negatively with l-arginine, but positively with putrescine. As the levels of l-arginine and putrescine were similar between the control and schizophrenia groups and there were no significant correlations between LEAPs with any of the neurochemical variables in the schizophrenia group, it is unlikely that the arginine metabolic profile changes observed are attributed to the antipsychotic drugs. We also observed a negative correlation between brain tissue pH and arginase activity and a positive correlation between PMI and l-arginine in the schizophrenia, but not in the control group. These findings indicate the importance of matching experimental conditions (such as the tissue pH and PMI) in human tissue research. Second, the present study investigated how brain arginine metabolism was affected in schizophrenia in the BA8 region only with 20 cases per group. It is therefore essential to validate the research findings in future with larger sample sizes and tissue from other brain regions, and to determine the disease specificity by comparing the profile pattern changes with other types of psychiatric disorders. Nevertheless, the present study extends previous work and provides further evidence of altered brain arginine metabolism in schizophrenia. The findings enhance our understanding of the pathogenesis of schizophrenia and may lead to the development of novel preventions and/or therapeutics for the disease.^65, 66^

Recently, many lines of research have suggested the involvement of the dysregulated polyamine system in suicidal behaviours.^67, 68, 69^ Surprisingly, we found lower arginase activities (~45% reduction, *P*<0.01) and a trend of reduced spermidine levels (~25% reduction) in the six suicidal cases when compared with the 14 non-suicidal schizophrenic cases (Supplementary Figure 1). Although the significant positive correlation between arginase activity and the age of disease onset was maintained in the non-suicidal schizophrenic cases, such relationship was not present in the suicidal cases (Supplementary Figure 1). As the sample size in the suicidal group was very small, future research is required to validate the findings with larger sample size and to understand the underlying mechanisms of these changes.

In summary, the present study demonstrates for the first time that changes of l-arginine metabolic enzymes and its downstream metabolites occur in the frontal cortex (BA8) in patients with schizophrenia. Specifically, we found increased levels of arginase activity, arginase II protein expression, agmatine tissue concentration and glutamate/GABA ratio, and reduced GABA level and eNOS protein expression in the schizophrenia cases. Although we replicated the GABAergic deficits in schizophrenia, the arginase and agmatine results are highly novel. The same pattern changes of agmatine in the brain (the present study) and blood^8^ in patients with schizophrenia merit future research to further understand its contribution to the development and pathogenesis of schizophrenia and to explore its potential as a biomarker of the disease.

## Figures and Tables

**Table 1:** Summary of demographic, testament and preservation data

*Diagnoses*	*Age (years)*	*Sex (M/F)*	*PMI (h)*	*pH*	*DI (years)*	*Age of onset (years)*	*Suicide (yes/no)*	*FRADD (Chl.Eq.)*	*LEAP*
Controls	57.75±3.21	11/9	41.23±3.48	6.31±0.06			0/20		
Schizophrenia	56.20±3.44	11/9	43.11±2.92	6.18±0.07	20.85±3.50	35.40±3.65	6/14	516±110	10.47±3.04
*P*-value	0.74	1	0.68	0.17					

Abbreviations: Chl.Eq, chlorpromazine equivalents; DI, duration of illness; F, female; FRADD, final recorded antipsychotic drug; LEAP, lifetime exposure to antipsychotic drugs; M, male; PMI, post-mortem interval.

Numeric data shown as mean±s.e.m.
