# Supplementary material for: Altered brain arginine metabolism in schizophrenia
Source: Transl Psychiatry. 2016 Aug 16;6(8):e871–. doi: 10.1038/tp.2016.144 (PMC5022089; doi:10.1038/tp.2016.144)
Supplement: Supplementary Figure 1 Legend [file tp2016144x2.docx]

**Supplementary Figure 1**

Mean (± SEM) levels of arginase activity (A) and spermidine (B) in the frontal cortex (BA8) from non-psychiatric control (n = 20), and non-suicidal (n = 14) and suicidal (n = 6) schizophrenia cases. Scattergrams showing correlations between arginase activity and the age of disease onset in the frontal cortex (BA8) from non-suicidal (C) and suicidal (D) schizophrenia cases. Asterisks indicate significant differences between groups at ** *p* < 0.01 or **** *p* < 0.0001.
