# Supplementary material for: Altered brain arginine metabolism in schizophrenia
Source: Transl Psychiatry. 2016 Aug 16;6(8):e871–. doi: 10.1038/tp.2016.144 (PMC5022089; doi:10.1038/tp.2016.144)
Supplement: Supplementary Table 1 [file tp2016144x3.docx]

**Supplementary Table 1:**

Demographic, tissue collection, pharmacological and experimental data from subjects with schizophrenia and non-psychiatric controls.

| **Diagnoses** | **Age** | **Sex** | **Sui** | **DI** | **pH** | **PMI** | **Cause of Death** | **FPAD** | **FRADD** | **LEAP** |
| --- | --- | --- | --- | --- | --- | --- | --- | --- | --- | --- |
|  | **(yr)** |  |  | **(yr)** |  | **(hr)** |  |  |  |  |
| **Controls** | 42 | M | N |  | 6.45 | 30.5 | Ishaemic Heart Disease (IHD) |  |  |  |
|  | 52 | M | N |  | 6.52 | 33.75 | Cardiomegaly and IHD, CAD |  |  |  |
|  | 47 | F | N |  | 5.89 | 24 | Pulmonary embolus |  |  |  |
|  | 75 | F | N |  | 6.01 | 53 | Multi organ failure, septicaemia, IHD |  |  |  |
|  | 70 | M | N |  | 6.11 | 59 | RV Rupture, IHD |  |  |  |
|  | 75 | M | N |  | 6.19 | 69.4 | Cardiogenic Shock, AMI |  |  |  |
|  | 55 | M | N |  | 6.69 | 30.5 | Coronary Artery Atherosclerosis |  |  |  |
|  | 66 | M | N |  | 6.47 | 71.75 | Coronary Artery Atheroma |  |  |  |
|  | 77 | F | N |  | 6.32 | 17 | Hypertensive heart disease |  |  |  |
|  | 63 | F | N |  | 6.55 | 50.25 | Coronary Artery Atherosclerosis |  |  |  |
|  | 56 | F | N |  | 5.88 | 24 | Pericardial Tamponade |  |  |  |
|  | 66 | F | N |  | 6.37 | 43 | Acute myocardial infarct (AMI) |  |  |  |
|  | 30 | M | N |  | 5.86 | 27 | Coronary Artery Atheroma |  |  |  |
|  | 38 | F | N |  | 6.26 | 52 | Pulmonary Thromboembolism |  |  |  |
|  | 71 | M | N |  | 6.33 | 50 | Ishaemic Heart Disease |  |  |  |
|  | 59 | F | N |  | 6.58 | 20.5 | Congestive Cardiac Failure |  |  |  |
|  | 62 | F | N |  | 6.45 | 40 | Ishaemic Heart Disease |  |  |  |
|  | 72 | M | N |  | 6.21 | 39 | Coronary Artery Atheroma |  |  |  |
|  | 42 | M | N |  | 6.61 | 43 | Coronary Artery Atheroma |  |  |  |
|  | 37 | M | N |  | 6.40 | 47 | Cardiomyopathy |  |  |  |
|  |  |  |  |  |  |  |  |  |  |  |
| **Schizophrenia** | 38 | M | Y | 11 | 6.44 | 36 | Hanging | Fluphenazine | 200 | 2.200 |
|  | 59 | M | N | 2 | 5.89 | 27.6 | Ischaemic Heart Disease | None recorded |  |  |
|  | 38 | F | Y | 17 | 6.43 | 20 | Burning | Fluphenazine | 485 | 8.240 |
|  | 79 | F | N | 1 | 6.27 | 26 | Hypothermia | Fluphenazine | 330 | 0.330 |
|  | 66 | M | N | 30 | 6.19 | 43.5 | Ischaemic Heart Disease, CA lung |  |  |  |
|  | 79 | M | N | 56 | 6.21 | 44 | Pneumonia | Risperidone | 800 | 44.800 |
|  | 58 | M | N | 36 | 6.63 | 42.5 | Ischaemic Heart Disease | Zuclopentixol | 506.25 | 18.225 |
|  | 61 | M | Y | 18 | 6.01 | 45.5 | Hypovolaemic shock, incised wrist | Risperidone | 285 | 5.130 |
|  | 76 | F | N | 22 | 6.28 | 52 | Aspiration of food bolus | None recorded |  |  |
|  | 58 | F | N | 8 | 5.85 | 72 | Pleural metastasis, CA of R breast | None recorded |  |  |
|  | 55 | F | N | 33 | 5.52 | 64 | Diabetic Ketoacidosis | Fluphenazine | 37 | 1.221 |
|  | 65 | F | N | 18 | 6.35 | 50 | Ruptured Abdominal Aneurysm | Fluphenazine  Haloperidol | 550 | 9.900 |
|  | 30 | M | Y | 10 | 6.65 | 19 | Hanging | Zuclopenthixol Amisulpride | 1575 | 15.750 |
|  | 34 | F | Y | 2 | 6.23 | 42 | Drowning | Risperidone | 1200 | 2.400 |
|  | 70 | M | N | 20 | 5.80 | 46 | Bonchpneumonia | None recorded |  |  |
|  | 59 | F | N | 35 | 6.19 | 44.5 | Respiratory Failure | Clozapine | 631.75 | 22.111 |
|  | 48 | F | N | 22 | 6.21 | 52.5 | Pulmonary Thromboembolism | Fluphenazine  Chlorpromazine | 700 | 15.400 |
|  | 71 | M | N | 53 | 6.45 | 48 | Aspiration of food bolus | Trifluoperazine | 150 | 7.950 |
|  | 42 | M | Y | 8 | 6.44 | 47 | Hanging | Haloperidol | 128 | 1.024 |
|  | 38 | M | N | 15 | 5.52 | 40 | Mediastinitis | Haloperidol | 160 | 2.400 |

**Abbreviations**: DI = duration of illness; F = female; FPAD = Final prescribed antipsychotic drug(s); FRADD = final recorded antipsychotic drug dose (mg chlorpromazine equivalents/day); Hr = hour; LEAP = lifetime exposure to antipsychotic drugs (mg chlorpromazine equivalents per year x 10^-3^); M = male; N = no; PMI = postmortem interval; Sui = suicide completion; Y = yes; Yr = year.
